# *In vitro* proliferation and differentiation of canine bone marrow derived mesenchymal stem cells over hydroxyl functionalized CNT substrates

**DOI:** 10.1016/j.btre.2019.e00387

**Published:** 2019-10-27

**Authors:** A.P. Madhusoodan, Kinsuk Das, Bhabesh Mili, Kuldeep Kumar, Ajay Kumar, A.C. Saxena, Praveen Singh, Triveni Dutt, Sadhan Bag

**Affiliations:** a Division of Physiology and Climatology, ICAR – Indian Veterinary Research Institute, Izatnagar, Uttar Pradesh, India; b Biochemistry and Food Science Section, ICAR – Indian Veterinary Research Institute, Izatnagar, Uttar Pradesh, India; c Division of Surgery, Izatnagar, ICAR – Indian Veterinary Research Institute, Uttar Pradesh, India; d Biophysics, Electron Microscopy and Instrumentation Section, ICAR – Indian Veterinary Research Institute, Izatnagar, Uttar Pradesh, India; e Division of Livestock Production and Management, ICAR – Indian Veterinary Research Institute, Izatnagar, Uttar Pradesh, India

**Keywords:** Canine MSCs, CNTs, Cellular behavior, Cytocompatibility, Differentiation

## Abstract

Nanotopography of culture substrate acts as a positive cue in cell-biomaterial based tissue regeneration. Considering the potentiality of carbon nanotubes (CNTs) this study was designed to evaluate its two functionalized form by an *in vitro* culture condition using canine mesenchymal stem cells as cellular model. Cells were isolated and its behaviour, proliferation and differentiation processes were elucidated onto CNT substrates. Beside the variations in cellular behaviour it was remarkably noted that even though proliferation was reduced but osteogenic and chondrogenic differentiation was enhanced over multi-walled CNTs, whereas neuronal differentiation was better supported by single walled CNTs as evidenced by our cytochemical, immunocytochemical, gene expression and flow cytometry assays. The former one was noticed more cytocompatible by our different apoptosis studies. The outcome of these experiments collectively indicated that hydroxylated functionalized CNTs could be a potential scaffold constituent for future experimentations as well as for the application in regenerative medicine.

## Highlights

• Carbon nanotubes have widely been explored as attractive scaffold component for promoting tissue regeneration.
• The importance of canine species in human drug trials explains its potentiality in experimental regenerative medicine.
• Hydroxyl functionalized CNTs substrate has not been tested with the perspective of its application in stem cell based tissue regeneration.
• The findings lead to exploit canine MSCs as a cellular model in experimental regenerative medicine and, (−OH) CNTs for its biological applications.

## Graphical abstract

**Figure:**
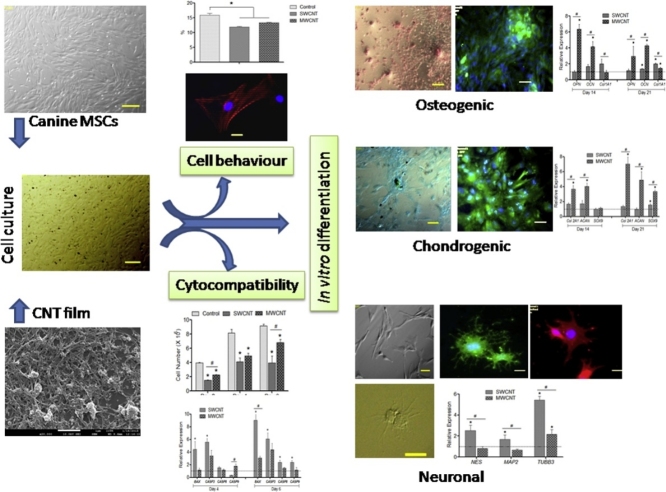

## 1 Introduction

Multipotent mesenchymal stem cells (MSCs) are extensively used in regenerative medicine for their valuable features of self-renewability and differentiation potentiality that give support to the regeneration of diseased tissues [1,2]. Behaviour of stem cell can be controlled effectively by manipulating the culture substrates [3]. Nanotopography, one of the regulatory biophysical cues has been shown to be an integral part of tissue regeneration in stem cell based therapy where cells and scaffolds are the two major components in successful making of engineered tissue [4]. Developments in nanotechnology have documented the potentiality of carbon nanotubes (CNTs) as scaffold component in tissue engineering application owing to their unique mechanical, chemical properties [5].

Among the animal models, dog (*Canis lupus familiaris*) exhibit nearly similar physiological and behavioural characteristics to human and, clinical trials of human drugs performed over dog propose that this species could also be a promising animal model in regenerative medicine [6,7]. In recent years, variety of nanomaterials like nanomorphous calcium phosphate, hydroxyapatite, chitosan, and alginate hydrogel have been tested on canine model for prospective tissue engineering applications [[8], [9], [10], [11]].In canines, MSCs can be isolated from sources like bone marrow, adipose tissue and umbilical cord components [[12], [13], [14]]. These cells also have therapeutic potentiality as evidenced in veterinary medicine [15]. Therefore, canine MSCs could be an alternative cellular model for experimental regenerative medicine as well as for the purpose of *in vitro* evaluation of various nanomaterials before their application. Recently, in this laboratory three different types of carboxyl and polyethylene glycol functionalized CNTs have been tested on canine MSCs aiming to categorize their suitability as scaffold component [16]. However, apart from few toxicity studies on human cell lines [17,18], hydroxyl (−OH) functionalized CNTs have been least explored for their applicability in biomedical sciences and not been tested in the area of cell biomaterial based tissue engineering. Therefore, the objective of this study was to find out such possibility using canine MSCs as cellular model. In this study, canine MSCs have been isolated from bone marrow, characterised, cultured and differentiated *in vitro* over two varieties of (−OH) functionalized CNT substrates. Different experiments have been conducted principally to observe the cellular behaviour, lineage specific differentiation potentiality of canine MSCs onto (−OH) CNTs, and also to evaluate the cytocompatibility of these substrates. Outcome of this study could lay a platform for *in vivo* application of (−OH) functionalized CNT scaffolds for stem cell based regenerative medicine and tissue engineering in future.

## 2 Materials and methods

### 2.1 Isolation of canine mesenchymal stem cells

Healthy dogs (*Canis lupus familiaris*) were utilized for the collection of bone marrow which was aseptically aspirated from iliac crest by complying with the guideline of Ethics Committee (IAEC). Mononuclear cells were first separated by density gradient centrifugation followed by plating with MSC culture medium (supplementary document) in tissue culture flask (Nunc, Germany) and maintained in a humidified incubator of 5% CO_2_ at 37^0^C. Cells were serially passaged by trypsinization method. We checked their spindle shaped morphology, plastic adherent property, immunophenotypic expression of MSC specific markers and also their lineage specific differentiation potential *in vitro*.

For immunophenotyping, the confluent culture was fixed in 4% paraformaldehyde in PBS for 20 min at room temperature (RT), washed with PBS, and permeabilized in 0.25% Triton X-100 in PBS for 15 min. After PBS washing, nonspecific binding was blocked with 2% bovine serum albumin (BSA) in PBS for 1 h at room temperature. Cells were incubated with MSC specific primary antibodies (positive for CD 73, CD 90, CD 105 and negative for CD 45; supplementary document) for overnight at 4^0^C. After washing with PBS, the cells were then incubated with corresponding secondary antibodies (supplementary document), for 4 h in darkness at room temperature. After PBS washing, culture substrate was counter stained with DAPI ProLong® Gold antifade solution (Invitrogen, USA). Images were captured in inverted fluorescence microscope (Carl Zeiss, Germany) with Axio Vision 4.0 image analysis system. To evaluate homogenecity of canine MSCs, fourth passage cells were analyzed by the use of flow cytometry. Aliquots containing 1 × 10^6^ cells for each marker was separately fixed, permeabilized blocked followed by incubation with primary and secondary antibodies as per the standard immunocytochemical staining protocol with the same combinations of antibodies used earlier for each marker. Flow cytometer (FACS Calibur, BD Bioscience, USA) settings were established using unstained cells. Cells were gated by forward scatter to eliminate debris. To eliminate the possible auto fluorescence of canine MSCs, contribution of unstained cells were removed in the measured channel. Data was analysed by recording 10,000 events with Cell Quest Pro software (BD Bioscience, USA). For tri-lineage differentiation, canine MSCs were maintained to get 70–80% confluence before replacing with differentiation medium. Briefly, the MSC medium was discarded and the wells were washed with PBS. Respective differentiation medium (supplementary document) was added in individual wells and maintained inside CO_2_ incubator. Mediums were refreshed on every 3rd day and maintained for 21 days for osteogenic, chondrogenic and adipogenic differentiations. Thereafter, the cultured cells were first washed with PBS and then fixed with 4% paraformaldehyde, stained with alizarin red, alcian blue and oil red-o for respective differentiations. After washing substrates were imaged by phase contrast microscope (Olympus, Japan). Passage 4 Canine MSCs were used for all further experimentation.

### 2.2 Fabrication of CNT substrates

For preparing CNT substrates, hydroxyl functionalized Single Walled Carbon Nanotubes (OH-SWCNTs; Sysco Research Laboratory, India; diameter 1–2 nm, length 5–30 μm) and Multi Walled Carbon Nanotubes (OH-MWCNTs; Sysco Research Laboratory, India; diameter 10–20 nm, length 10–30 μm) were dispersed separately in ethanol (0.1 wt %) by 4 h of sonication to get proper dispersion and homogeneity. Suspension was spray coated over pre-heated round coverslips to make a thin coating of CNTs. These CNT substrates were UV sterilized prior to cell culture. To visualize the surface topography, Field Emission Scanning Electron Microscopic (FESEM; Zeiss, Germany) imaging at an accelerating voltage of 10 kV was done.

### 2.3 Cell behaviour over CNT substrates

Cells were cultured at a density of 1000cells/cm^2^ in tissue culture plate as control and also onto CNT substrates. Cell spreading area was measured with Image J software after imaging different fields on day 2, 4 and 6 of the experiment.

We immunostained these cells to visualize the difference if any in the orientation of F-actin filament. Cultured cells were fixed with 4% paraformaldehyde (PFA), permeabilized by TritonX-100 and blocked for nonspecific site using 2% bovine serum albumin (BSA). The immunostaining with Alexa Fluor® 680 conjugated phalloidin was followed by nuclear staining with DAPI ProLong® Gold antifade solution (both Invitogen, USA).

Further we checked the colony forming ability of the cells cultured over CNT substrates. Cells were seeded at low density of 50cells/cm^2^ over control as well as coated coverslips placed in 6-well culture plates and maintained for 14 days. After paraformaldehyde fixation the culture plates were stained by 0.5% crystal violet solution. The colonies showing more than 20–30 cells were counted over the control and CNT substrates.

### 2.4 Cytocompatibility evaluation of CNT substrates

We performed three different experiments to evaluate the cytocompatibility of these CNT substrates. Cells cultured in the culture plate without any CNT coated coverslips were taken as control.

Cytocompatibility was assessed by the changes observed in the rate of cell proliferation onto the CNT substrates. Cells were plated at a density of 1 × 10^4^/cm^2^ both onto control and substrates. Metabolically active cell number was quantified at different days of culture by 3-(4,5-dimethylthiazol-2-yl)-2,5-diphenyltetrazolium bromide (MTT) assay (Invitrogen kit, USA).

We checked the expression changes in different genes associated with cell apoptosis. Total RNA was extracted from the cultured cells by TRIzol® reagent (Invitrogen, USA) and complementary DNA (cDNA) was synthesised using iScript™ cDNA Synthesis Kit (Bio-Rad, USA). Reverse transcriptase-polymerase chain reaction (RT-PCR) and semi-quantitative gene expression analysis was carried out by Real-Time PCR system (Bio-Rad, USA) using SsoFast™ EvaGreen® Supermix kit (Bio-Rad, USA) with the canine specific primers for the apoptosis associated genes like *BAX*, *CASP3*, *CASP8* and *CASP9*. The *GAPDH* was taken as house keeping control gene. Among these, primers of CASP8 and CASP9 have been designed by DNA star software (supplementary document). Changes in expression of different transcripts were calculated and represented in terms of fold change with respect to cells cultured on control plate [19].

Further we quantified the apoptotic and necrotic cells onto CNT substrates. Flow cytometry assay was done by using Annexin V- FITC/PI Apoptosis Detection Kit (BioVision, USA) in a FACS Calibur (BD Bioscience, USA). Data was analysed with Cell Quest Pro software (BD Bioscience, USA).

### 2.5 *In vitro* differentiation over CNT substrates

Canine MSCs were cultured both on CNTs and control (culture wells without CNT coated coverslips), at the density of 12500/cm^2^ and maintained for 3 days to get 70–80% confluence before replacing with differentiation medium. Briefly, the MSC medium was discarded and the wells were washed with PBS. Respective differentiation medium (supplementary document) was added in individual wells and maintained inside CO_2_ incubator. Mediums were refreshed on every 3rd day and maintained for 21 days for osteogenic and chondrogenic differentiations. In case of neuronal differentiation 24 h prior to neuronal induction cells were bathed with pre-induction medium followed by switching over to induction medium (supplementary document) for another six days. Medium was refreshed on every 3rd day.

#### 2.5.1 Cytochemical staining

After 21 days of osteogenic and chondrogenic differentiation, cultured cells were first washed with PBS and then fixed with 4% paraformaldehyde, stained with alizarin red and alcian blue respectively. After washing substrates were imaged by phase contrast microscope (Olympus, Japan).

#### 2.5.2 Differentiation associated gene expression

On day 14 and 21 of osteogenic and chondrogenic differentiations and, after 6 days of post induction in case of neuronal differentiation total RNA was extracted and was reverse transcribed for cDNA synthesis. RT-PCR and semi– quantitative gene expression study for the respective experiments was carried out by Real-Time PCR system (Bio-Rad, USA) with canine specific primers of osteocyte specific genes *BGLAP*, *SPP1* and *COL 1A1*; chondrocyte specific genes *Col 2A1*, *ACAN* and *SOX9*; and neuron specific genes *TUBB3*, *MAP2* and *NES*. *GAPDH*was considered as the endogenous house keeping control gene. Among these, primer of MAP2 has been designed by DNA star software (supplementary document). Fold changes of in transcript expression were calculated by the method proposed by Pfaffl [19] with respect to cells differentiated onto control plate without any CNT substrate.

#### 2.5.3 Immunophenotyping of differentiated cells

At the end of differentiation experiments immunocytochemistry was done to detect osteocalcin for osteocytes and aggrecan for chondrocytes localization in control and CNT substrates. After neuronal induction, we immunostained the differentiated cells with neuron specific markers like β-III tubulin and MAP2.

To quantify the differentiated cells and to express it in terms of percentage positive cells compared to control flow cytometry assay was done. Cultured cells were harvested and immunostained by incubation with same set of antibodies used for immunocytochemistry. Data acquisition was done in flow cytometer (FACS Calibur, BD Bioscience, USA).

### 2.6 Statistical analysis

All quantitative data was analyzed by one way ANOVA, followed by Duncan *Post hoc test* (SPSS Inc, USA) and expressed as mean ± standard error of the mean. Statistical significance was considered at p < 0.05 for all the experiments.

## 3 Results

### 3.1 Isolation, culture and characterization of canine MSCs

**Fig. 1:**
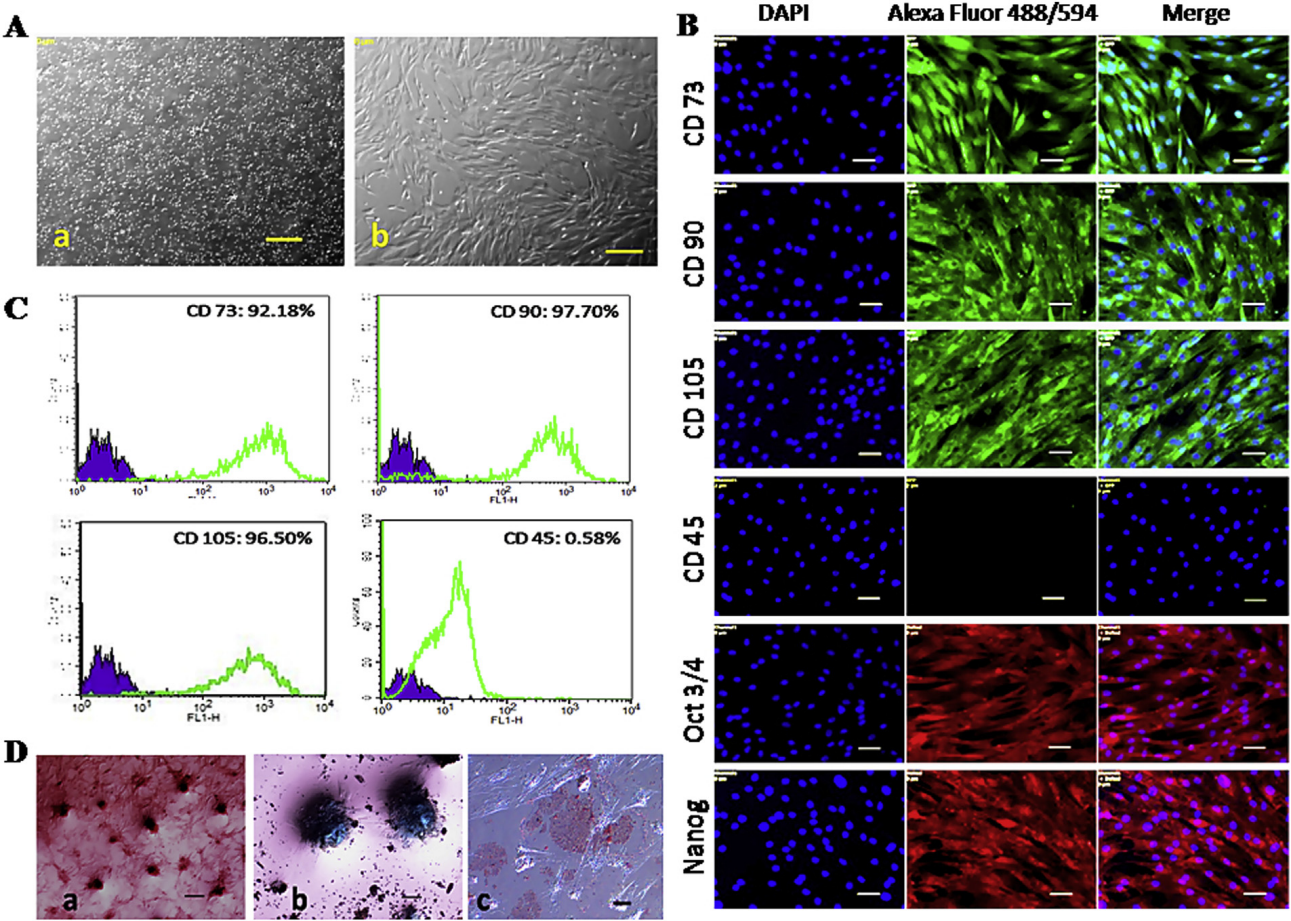
Characterization of canine mesenchymal stem cells (MSCs). **(A**) Canine MSCs. (a) bone marrow mono nuclear cells and (b) fibroblastic morphology in P3. Scale: 100 μm **(B)** Immunostaining with MSC surface markers and pluripotency markers. Scale: 50 μm. **(C)** Flow cytometry of surface markers **(D)** Cytochemical staining after *In vitro* differentiation into (a) alizarin red, scale: 100 μm (b) alcian blue, scale: 100 μm and (c) Oil red-O, scale: 50 μm.

The bone marrow derived cells were round in shape initially but, as the day progressed some of the cells became adherent and afterwards attained the spindle shaped morphology (Fig. 1A). Cells were able to maintain this morphology in succeeding passages. Immunophenotyping of cell monolayer showed positive for CD73, CD90, CD105 while negative for CD45, also expressed pluripotency markers Nanog and Oct4 (Fig. 1B). Quantification of surface markers by flow cytometry revealed that 92.18% cells were positive for CD73, 97.7% for CD90, 96.5% for CD105 and 0.58% were positive for CD45 indicating that the cell population utilized in this study were predominantly the MSCs (Fig. 1C). Cells were able to differentiate into osteocytes, chondrocytes and adipocytes under standard *in vitro* differentiation. Osteogenic differentiation was evident by presence of mineralized aggregates in Alizarin Red staining. Proteoglycan accumulation was evidenced by alcian blue staining after chondrogenic differentiation. Adipocyte differentiation was confirmed by presence of lipid droplets in Oil Red O staining (Fig. 1D).

### 3.2 Characterization of CNT substrates

**Fig. 2:**
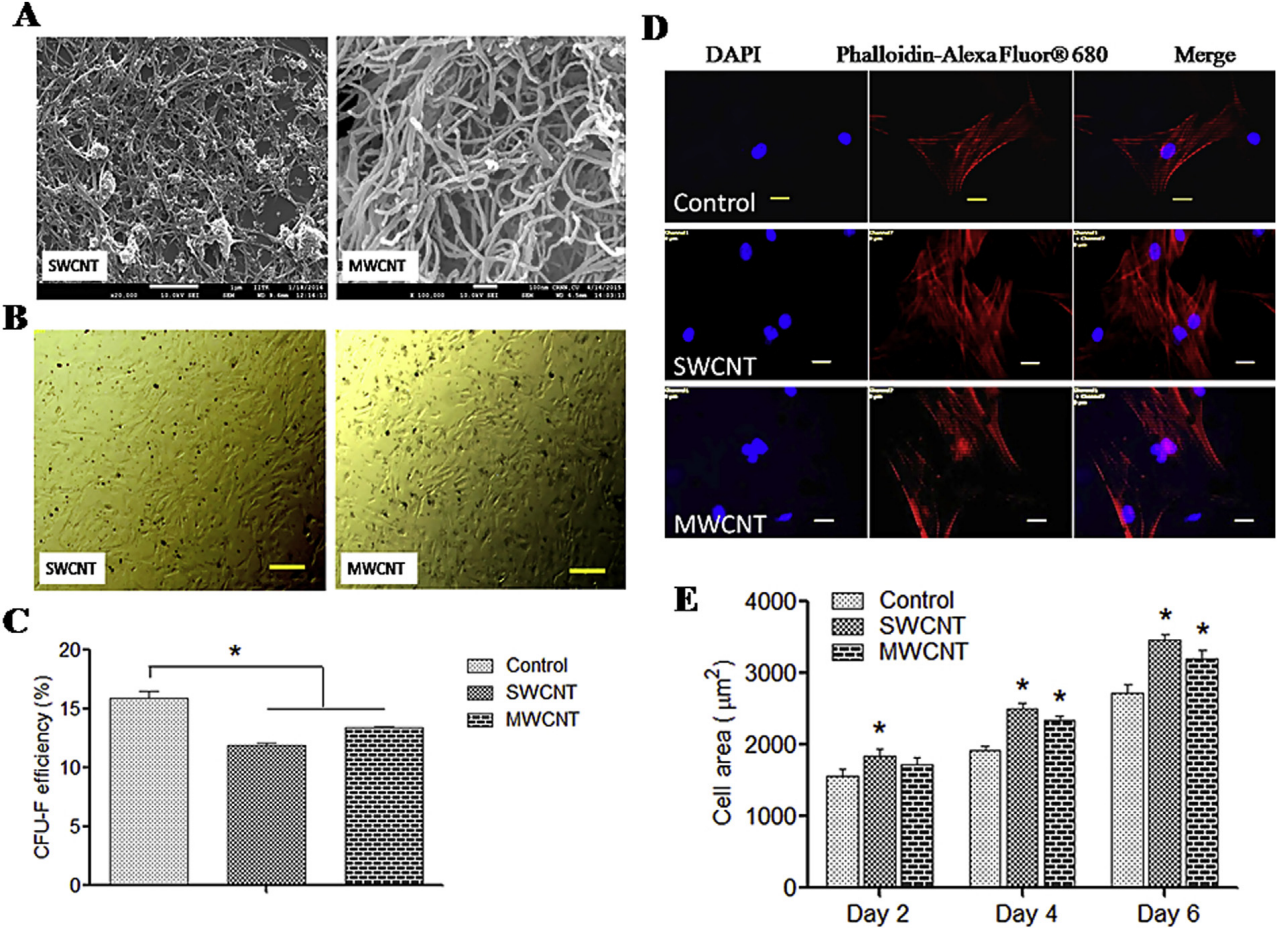
Characterization of CNT substrate and cellular behaviour study. **(A)** FSEM images of two types of CNT films. **(B)** P4 cells on control and CNT films. Scale bars: 100 μm. **(C)** Colony forming assay on control and CNT films. **(D)** Immunostaining of actin filament. Scale bars: 20 μm. **(E)** Cell spreading area at different time intervals. The symbols * indicate significance (*P* < 0.05) with respect to control (n=30).

Substrates were prepared over coverslip and visualized for topography. FESEM images indicated that a layer of CNTs were formed by both the types and individual nanotubes were notable (Fig. 2A).

### 3.3 Cellular behaviour study

Nano feature over the cover slip evidenced by SEM images could be helpful for the cells to interrelate at the cell-material interface. To check its effect, cellular behaviours such as cell area, colony forming assay as well as stress filament was evaluated. Canine MSCs proliferated well while maintaining their fibroblastic morphology both in control and CNT substrates (Fig. 2B). Subpopulations of cells were also capable to generate new colonies onto the CNT culture substrates. The ability of canine MSCs to form colony was notably lower on both SWCNT and MWCNT substrates compared to control (Fig. 2C) but cell spreading area was noticed significantly higher initially over SWCNT scaffold compared to control and MWCNT scaffold (Fig. 2E). By immunocytochemical staining we evidenced discrete stress fiber bundles in treatment groups which seems more prominent as well as oriented parallel with each other as compared to control (Fig. 2D).

### 3.4 Cytocompatibility of CNT substrates

**Fig. 3:**
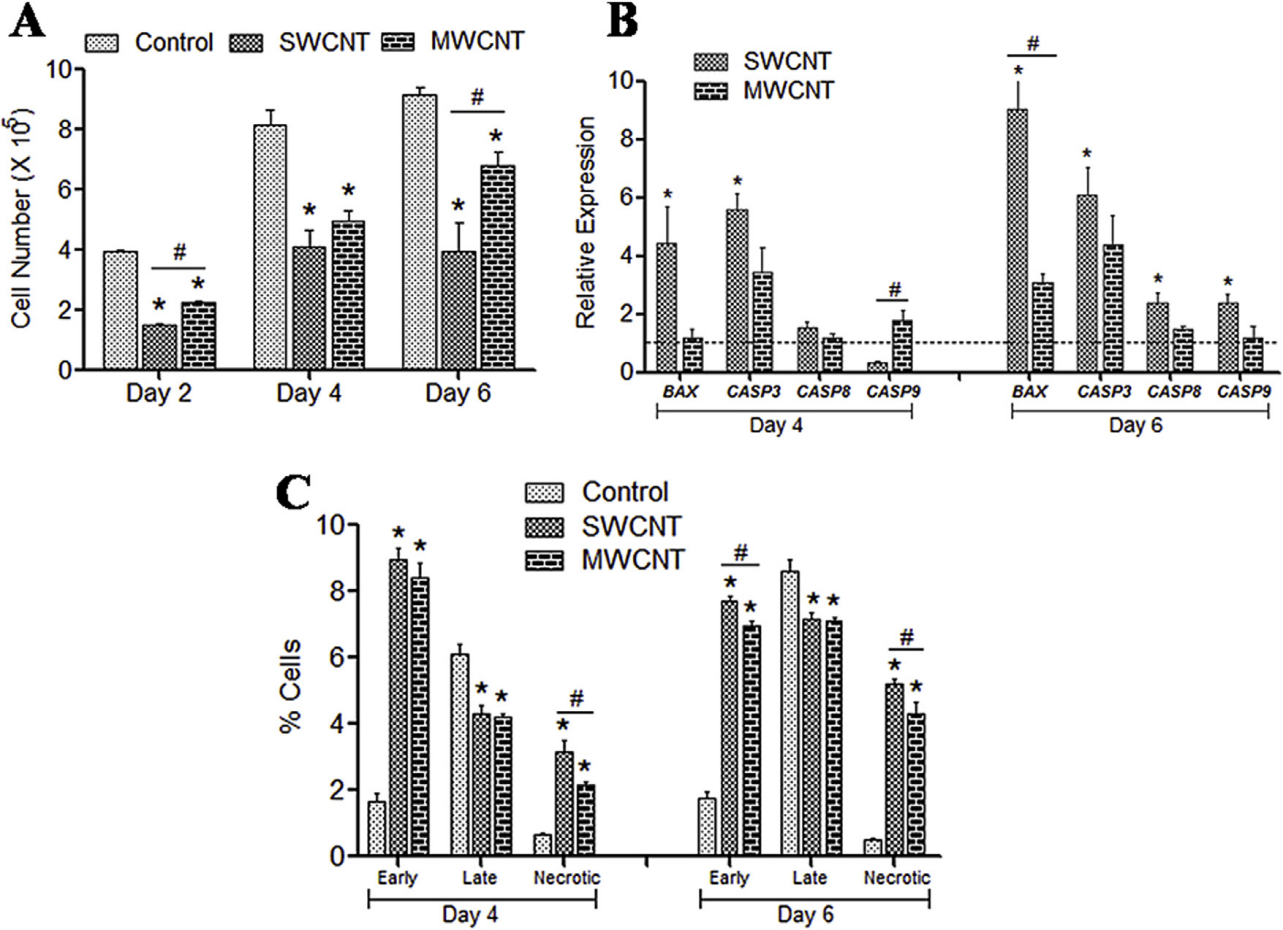
Cytocompatibility study. **(A)** Cell proliferation study by MTT assay of canine MSCs cultured on control and different CNT films. **(B)** Gene expression study. Dashed line indicates values of target genes in control conditions. **(C)** Annexin V–PI flow cytometry assay. For all qualitative experiments the symbols * and # indicate significance (*P* < 0.05) with respect to control and between the films, respectively, on a particular day.

MTT assay indicates the biocompatibility of culture substrates with respect to the proliferative behaviour of cells. Cell number decreased significantly on both the CNT surfaces (Fig. 3A). Among the OH functionalized CNTs, cell proliferation was found better in MWCNT compared to SWCNT throughout the experiment indicating MWCNT as less cytotoxic to canine MSCs.

Relative expression of apoptosis associated genes were studied upto 6^th^ day of culture (Fig. 3B). Expressions of *BAX* and *CASP3* were always noticed considerably higher on SWCNT as compared to control and MWCNT substrates. However, the *CASP9* was noticed high on MWCNT substrate initially but together with *CASP8* it was found significantly high only in SWCNT group after day 6. It is to be noted that *BAX, CASP3* and *CASP8* were never found considerably high in the cells cultured over MWCNT substrates.

These results were found in accordance with our flow cytometry assay of AnnexinV-PI stained cells during culture period. The percentages of early apoptotic as well as necrotic cells were noticed significantly higher on both types of CNT substrates upto day 6 of culture. However, a higher percentage of cells were found necrotic over SWCNT substrate (Fig. 3C).

### 3.5 *In vitro* differentiation study

#### 3.5.1 Osteogenic differentiation

**Fig. 4:**
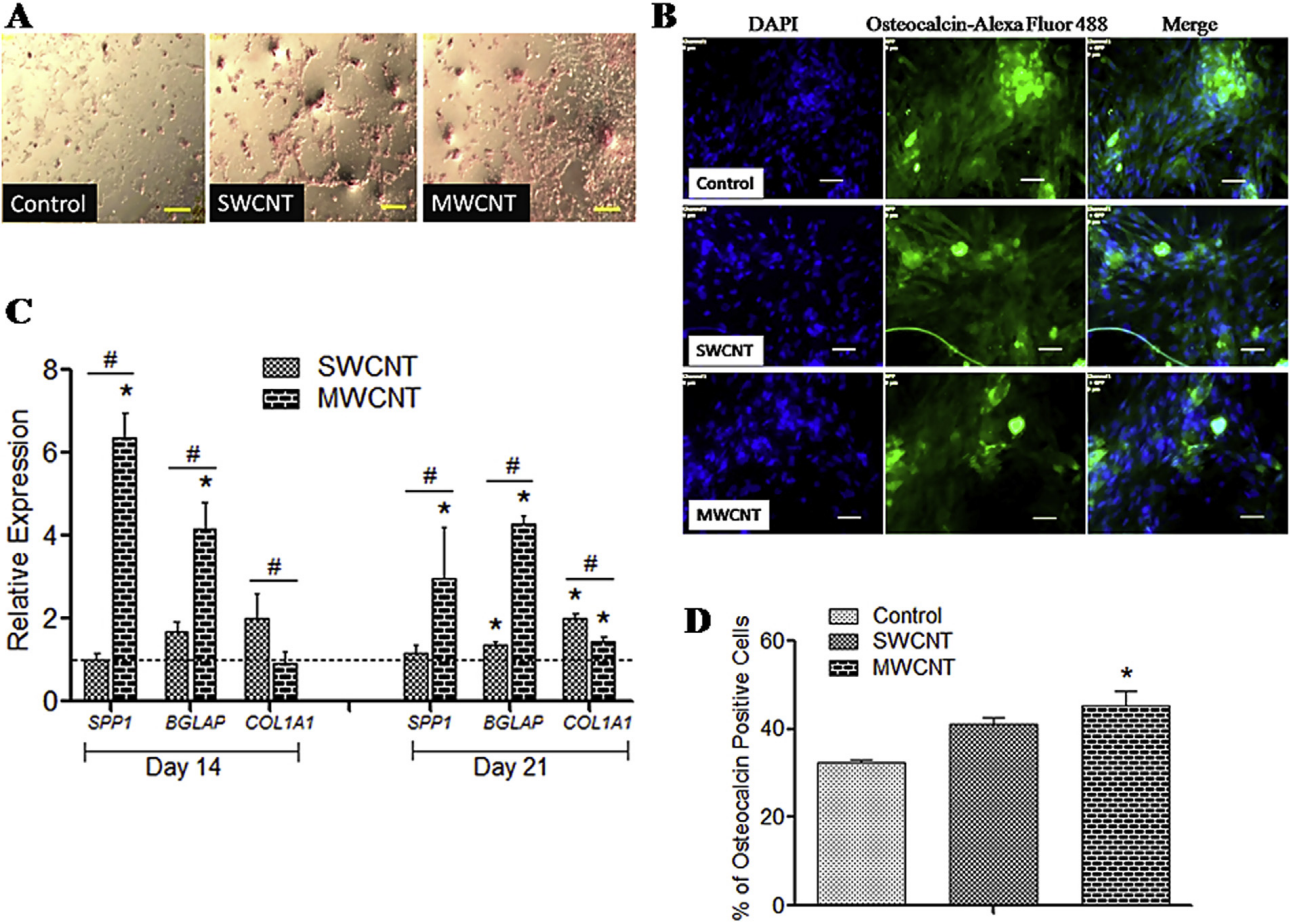
Osteogenic differentiation of canine MSCs. **(A)** Alizarin red staining over control and CNT films. Scale-100 μm. **(B)** Immunofluorescence of osteocalcin positive cells. Scale-50 μm. **(C)** Relative gene expression study. Dashed line indicates values of target genes in control conditions. (**D**) Flow cytometry of osteocalcin-positive cells. The symbols * and # indicate significance (*P* < 0.05) with respect to control and between the films, respectively for the qualitative experiments.

Alizarin Red staining after 21 days of osteogenic induction of canine MSCs showed some deposition of mineralized aggregates in control and CNT scaffolds. Although not quantified, an apparently higher number, size and darker nodules were noticed over MWCNT substrate (Fig. 4A). We checked the relative expression profile of osteocyte specific genes like *BGLAP*, *SPP1*and *Col1A1*. Expression profile of *BGLAP* and *SPP1* was always found significantly higher in MWCNT, while *ColIA1* in case of SWCNT (Fig. 4C). Immunostaining with anti-osteocalcin antibody confirmed the differentiation on both control and CNT (Fig. 4B). On flow cytometry analysis significantly higher population of *BGLAP* positive cells were noticed onto the MWCNT substrates (Fig. 4D).

#### 3.5.2 Chondrogenic differentiation

**Fig. 5:**
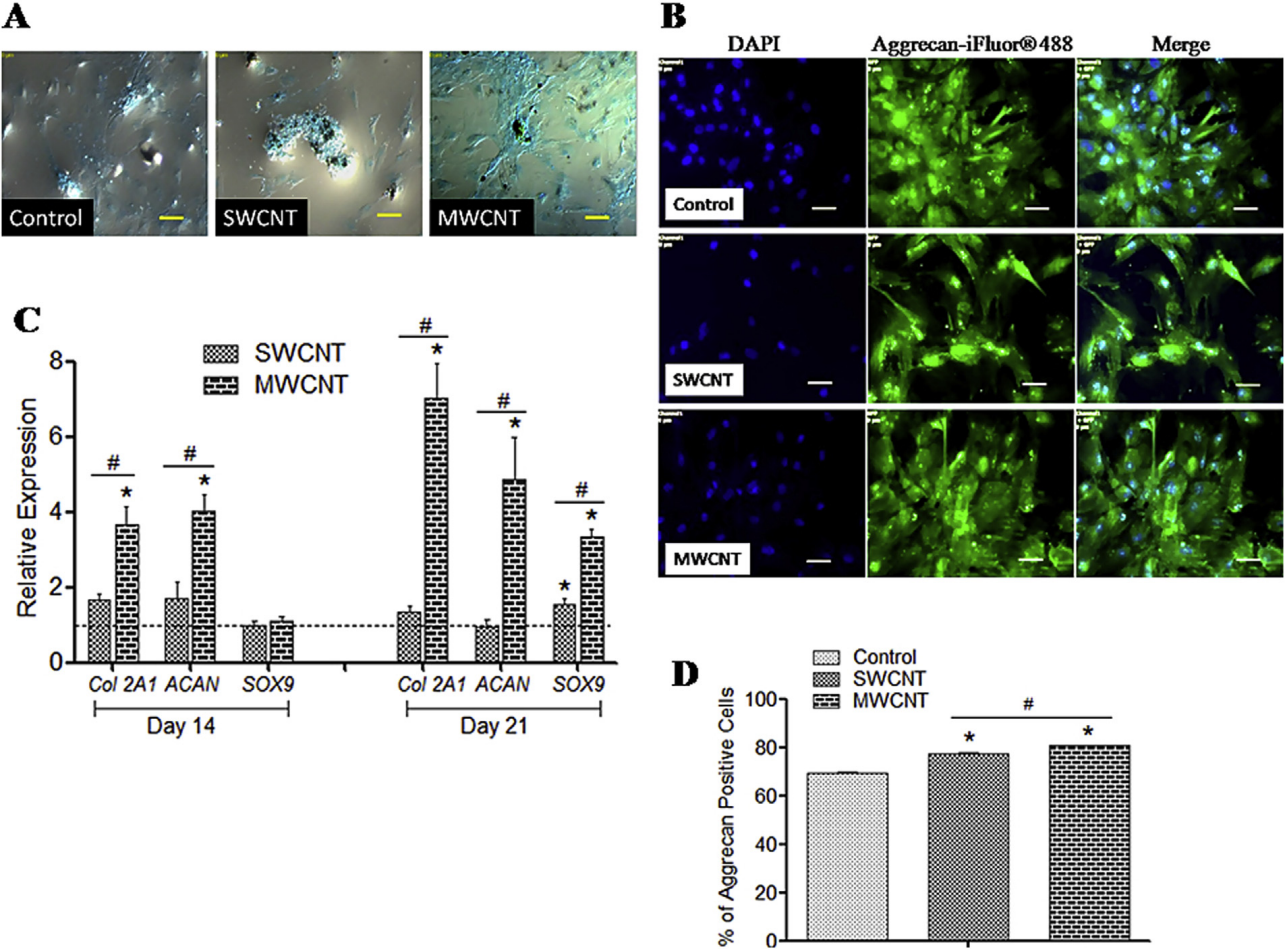
Chondrogenic differentiation of canine MSCs. **(A)** Alcian blue staining over control and CNT films. Scale-100 μm. **(B)** Immunofluorescence of aggrecan positive cells. Scale bars: 50 μm. **(C)** Relative gene expression study. Dashed line indicates values of target genes in control conditions. **(D)** Flow cytometry of aggrecan positive cells. The symbols * and # indicate significance (*P* < 0.05) with respect to control and between the films, respectively for the qualitative experiments.

Alcian Blue staining of chondrogenic culture showed proteoglycan aggregates in both control and CNT substrates. Apparently, larger aggregateswere noticed over MWCNT (Fig. 5A). Immunostainingby anti-aggrecan antibody established the differentiation both onto control and CNT (Fig. 5B). Time dependent relative expression profiles of chondrogenesis associated genes *ACAN*, *Col 2A1 and SOX9* revealed significant higher expression over MWCNT substrates (Fig. 5C). Significantly higher number of aggrecan positive cells was found over MWCNT substrate as evidenced by flow cytometry (Fig. 5D).

#### 3.5.3 Neuronal differentiation

**Fig. 6:**
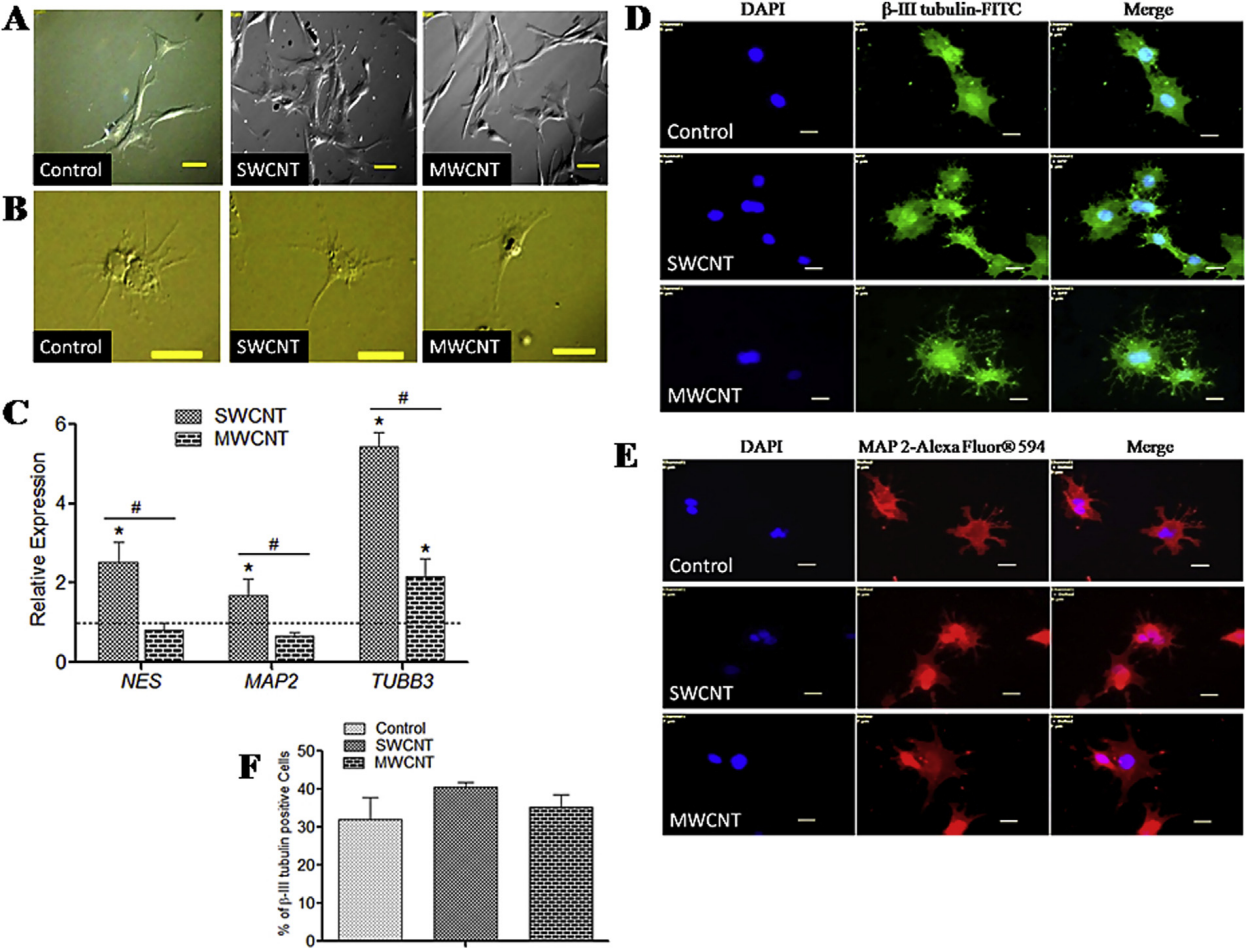
Neuronal differentiation of canine MSCs. **(A)** MSCs after 24 h of preinduction. Scale bars: 50 μm. **(B)** After 6 days of induction. Scale bars: 20 μm. **(C)** Relative gene expression study. Dashed line indicates values of target genes in control conditions. **(D)** Immunofluorescence of β-III tubulin-positive cells and (**E**) MAP2-positive cells. Scale bars: 20 μm. (**F**) Flow cytometry assay of β-III tubulin-positive cells. The symbols * and # indicate significance (*P* < 0.05) with respect to control and between the films, respectively for the qualitative experiments.

We noticed some morphological changes of canine MSCs during neuronal differentiation process. Changes in fibroblastic to a neuron like morphology and with multiple branching was noticed (Fig. 6A, B). Relative expressions of neuron specific genes *TUBB3*, *MAP2* and *NES* were significantly higher in SWCNT compared to control and MWCNT (Fig. 6C). The immunophenotypic expression of β III-tubulin and MAP2 confirmed the neuronal differentiation process (Fig. 6D, E). Flow cytometry analysis of β-Tubulin III positive cells indicated that canine MSCs cultured over SWCNT yielded more number of differentiated cells compared to control and MWCNT substrates (Fig. 6F).

## 4 Discussion

Fibroblast like morphology, expression of MSC specific surface markers, *in vitro* differentiation ability and non expression of haemopoietic stem cell markers led us to consider that the cell population utilized in this study were of MSCs.

### 4.1 Cellular behaviour study

Although the capability to form colonies was less but the evidence of larger spreading area of the MSCs cultured over CNT might be due to the dissimilarity in cytoskeleton orientation caused by the CNT surface. Influence of CNTs on hMSC morphology and its greater spreading area has been noticed earlier [20,21]. Later in our experiment we found an improved differentiation of MSCs which might be due tothe higher cell spreading leading to enhanced mechanotransduction process [22].

Stress fibre abundance and organization are modulated by mechanical stress cell encounters at cell-biomaterial interface [23].The parallel orientation of actin filament network might due to the cells’ attempt to resist their additional deformation over the CNT culture substrates [24]. From overall cellular behaviour study, we hypothesised that better cell spreading, organized stress filament abundance have been noticed as canine MSCs had a positive topographical cue for *in vitro* culture.

### 4.2 Cytocompatibility of CNT substrates

Cell needs more energy in adopting any nanomaterial surface resulting in a drop of their proliferation rate which is also common to the stem cells of different species over CNT culture substrates [20,[25], [26], [27], [28]]. Moreover, stem cell proliferation rate is generally reduced after the initiation of differentiation process. It has been reported that human MSCs (hMSCs) may differentiate early when cultured on fabricated nanocomposites including CNT substrates [21,29]. Although the rate of proliferation was slow but there was no evidence of decline in cell number indicating that CNTs do not have considerable cytotoxic effect on canine MSCs.

*CASP8* and *CASP9* expression are influenced by the extracellular and intrinsic cues respectively whereas both in a combined way trigger the executioner *CASP3* [30]. Non significant variation in *CASP8* expression on the MWCNT substrate might be the indication that the surface did not affect adversely to cause apoptosis. However, we noticed an initial higher expression of *CASP9* on MWCNT which might be the consequence of CNT’s cellular internalization capacity [31].

Categorizing CNTs as “toxic” is debatable as its effect on cell varies on application and cell type [32]. Size of CNTs and its dispersing ability in culture media have been reported to be the influencing factors in considering cytotoxicity [31,33,34]. Moreover, CNTs were found more biocompatible when present as a scaffold component because of less endocytosis by the cells [[35], [36], [37], [38]]. Liu et al. [18], in their study on a human cell line found that hydroxylation of MWCNTs reduces its cytotoxic effect by limiting the activation of mitochondrial mediated apoptotic pathway. Results established in these experiments illustrate the potentiality of OH-MWCNT as low cytotoxic substrate.

### 4.3 *In vitro* differentiation study

Much effort has been given to study the molecular mechanisms of interactions between stem cells and the anchoring nanomaterial substrate. In brief, mechanosensors of cell membrane and cytoplasmic transducers are primarily responsible for the modulation of stem cell proliferation and differentiation. Therefore, the mechanotransduction process is considered as the governing factor in modulating the behaviour of stem cells when cultured onto nanomaterial scaffolds [22,39].

The *SPP1* is upregulated at the initial mineralization phase of osteogenesis while *BGLAP* is considered asthe specific gene for osteoblast differentiation and helps to accumulate minerals in [40,41]. Further, Type-I collagen (*COL1A1*), is an ECM protein influence cellular behaviour at the time of differentiation [42]. In our experiment, significant expression of *BGLAP* and *SPP1* might be the indication of better osteogenesis leading to the generation of osteoblastic cells in MWCNT substrates. Better mineralization noticed by alizarin red staining, more osteocalcin-positive cells in flow cytometry onto MWCNT are in accordance with gene expression experiment. Several factors like topography of the culture substrate and its roughness, better cell spreading and tensile strength on actin filament have positive influences over osteogenesis [[43], [44], [45], [46]]. Improved osteogenesis of MSCs has been reported in many of the studies on different species [20,26,[46], [47], [48]].We could able to identify MWCNT substrate as a cue in acceleration of osteogenic differentiation process in canine MSCs.

The expression of *ACAN* and *Col 2A1*, the integral cartilage ECM components were found to be enhanced on MWCNT substrate might be due to the surface modification by CNTs. Charged nature of COOH in CNTs might augment *Col 2A1* expression leading to the modulation of chondrocyte biology [49]. Human MSCs cultured for chondrogenic differentiation over SWCNT scaffolds enhanced GAG synthesis within 2 weeks whereas such effect slightly decreased when cultured in a medium suspended with CNT particles [50]. Herein MWCNT substrates have enhanced chondrogenesis process compared to SWCNT as evidenced by gene expression study and flow cytometry assay of aggrecan positive cells.

CNTs have been explored widely as culture substrates for neuronal growth and differentiation because these nanotubes accelerate the onset of neuronal electrical activity, which directly interferes with neuronal signalling [51]. Spontaneous neuronal differentiation of human and rat bone marrow-derived MSCs on CNT substrates have also been reported [52,53]. In our study, neuron-like morphological changes and immunopositivity for β-III tubulin and MAP2 indicate that the MSCs had differentiated into neurons on the CNT substrates. In terms of our gene expression study canine MSCs differentiated better towards mature neurons on the SWCNT. More in number of β-III tubulin-positive cells on SWCNT as assessed by flow cytometry was in agreement with our gene expression findings. These results suggest that OH functionalized SWCNT based substrates might be considered as a promoter for stem cell differentiation toward the neuronal lineage.

## 5 Conclusions

In this study, we evaluated the effect of hydroxylated functionalized CNT substrates on canine bone marrow-derived MSCs considered as an *in vitro* cellular model. CNT surface induced slower proliferation rate, lower colony forming ability with changes in cytoskeleton orientation of the cells. MWCNT was noticed to be better cytocompatible with respect to proliferation and apoptosis studies and by the proper selection of CNTs it is possible to accelerate the stem cell differentiation. The differentiation studies suggest that OH-MWCNT has positive influence on osteogenic and chondrogenic differentiation of canine MSCs, while neuronal differentiation can be promoted by using OH-SWCNT substrate. The findings of our study will lead to comprehend the variations in behavior and differentiation potentiality of canine MSCs of on CNT substrates which could be the essential information for its future application in regenerative medicine.

## Declaration of Competing Interest

Authors declare no conflict of interest.

## References

[1] Jiang Y., Jahagirdar B.N., Reinhardt R.L., Schwartz R.E., Keene C.D., Xilma R., Ortiz-Gonzalez, Reyes M., Lenvik T., Lund T., Blackstad M., Du J., Aldrich S., Lisberg A., Low W.C., Largaespada D.A., Verfaillie C.M. (2002). Pluripotency of mesenchymal stem cells derived from adult marrow. Nature.

[2] Erices A., Conget P., Minguell J.J. (2000). Mesenchymal progenitor cells in human umbilical cord blood. Br. J. Haematol..

[3] Lee J.R., Ryu S., Kim S., Kim B.S. (2015). Behaviors of stem cells on carbon nanotube. Biomed. Res..

[4] Williams D.F. (2009). On the nature of biomaterials. Biomaterials.

[5] Thorstenson E.T., Ren Z., Chou T.W. (2001). Advances in science and technology of carbon nanotubes and their composites: a review. Compos. Sci. Technol..

[6] Tsai K.L., Clark L.A., Murphy K.E. (2007). Understanding hereditary diseases using the dog and human as companion model systems. Mamm. Genome.

[7] Schneider M.R., Wolf E., Braun J., Kolb H.J., Adler H. (2008). Canine embryo-derived stem cells and models for human diseases. Hum. Mol. Genet..

[8] Mostafa A.A., Zaazou M.H., Chow L.C., Mahmoud A.A., Zaki D.Y., Basha M., Hamid M.A. Abdel, Khallaf M.E., Sharaf N.F., Hamdy T.M. (2015). Injectable nanoamorphouscalcium phosphate based in situ gel systems for the treatment of periapicallesions. Biomed. Mater..

[9] Du B., Liu W., Deng Y., Li S., Liu X., Gao Y., Zhou L. (2015). Angiogenesis and bone regeneration of porous nano-hydroxyapatite/coralline blocks coated with rhVEGF165 in critical-size alveolar bone defects in vivo. Int. J. Nanomed..

[10] Zeng Y., Chen C., Liu W., Fu Q., Han Z., Li Y., Feng S., Li X., Qi C., Wu J., Wang D., Corbett C., Chan B.P., Ruan D., Du Y. (2015). Injectable microcryogels reinforced alginate encapsulation of mesenchymal stromal cells for leak-proof delivery and alleviation of canine disc degeneration. Biomaterials.

[11] Zang S., Dong G., Peng B., Xu J., Ma Z., Wang X., Liu L., Wang Q. (2014). A comparison of physicochemical properties of sterilized chitosan hydrogel and its applicability in a canine model of periodontal regeneration. Carbohydr. Polym..

[12] Vieira N.M., Brandalise V., Zucconi E., Secco M., Strauss B.E., Zatz M. (2010). Isolation, characterization and differentiation potential of canine adipose-derived stem cells. Cell Transplant..

[13] Lee K.S., Nah J.J., Lee B.C., Lee H.T., Lee H.S., So B.J., Cha S.H. (2013). Maintenance and characterization of multipotentmesenchymal stem cells isolated from canine umbilical cord matrix by collagenase digestion. Res. Vet. Sci..

[14] Kisiel A.H., McDuffee L.A., Masaoud E., Bailey T.R., Esparza Gonzalez B.P., Nino-Fong R. (2012). Isolation, characterization, and in vitro proliferation of canine mesenchymal stem cells derived from bone marrow, adipose tissue, muscle, and periosteum. Am. J. Vet. Res..

[15] DeBakker E., VanRyssen B., DeSchauwer C., Meyer E. (2013). Canine mesenchymal stem cells: state of the art, perspectives as therapy for dogs and as a model for man. Vet..

[16] Das K., Madhusoodan A.P., Mili B., Kumar A., Saxena A.C., Kumar K., Sarkar M., Singh P., Srivastava S., Bag S. (2017). Functionalized carbon nanotubes as suitable scaffold materials for proliferation and differentiation of canine mesenchymal stem cells. Int. J. Nanomed..

[17] Li Z., Liu T., Long J.M., Wu Y., Yan B., Ma P., Cao Y. (2019). The toxicity of hydroxylated and carboxylated multi-walled carbon nanotubes to human endothelial cells was not exacerbated by ER stress inducer. Chin. Chem. Lett..

[18] Liu Z., Liu Y., Peng D. (2014). Hydroxylation of multi-walled carbon nanotubes reduces their cytotoxicity by limiting the activation of mitochondrial mediated apoptotic pathway. J. Mater. Sci. Mater. Med..

[19] Pfaffl M.W. (2001). A new mathematical model for relative quantification in real-time RT-PCR. Nucleic Acid Res..

[20] Tay C.Y., Gu H., Leong W.S., Yu H., Li H.Q., Heng B.C., antang H.T., Loo S.C.J., Li L.J., Tan L.P. (2010). Cellular behavior of human mesenchymal stem cells cultured on single-walled carbon nanotube film. Carbon.

[21] Deligianni D.D. (2014). Multiwalled carbon nanotubes enhance human bone marrow mesenchymal stem cells’ spreading but delay their proliferation in the direction of differentiation acceleration. Cell Adh. Migr..

[22] Zhao C., Tan A., Pastorin G., Ho H.K. (2013). Nanomaterial scaffolds for stem cell proliferation and differentiation in tissue engineering. Biotechnol. Adv..

[23] Fletcher D.A., Mullins R.D. (2010). Cell mechanics and the cytoskeleton. Nature.

[24] Storm C., Pastore J.J., MacKintosh F.C., Lubensky T.C., Janmey P.A. (2005). Nonlinear elasticity in biological gels. Nature.

[25] Namgung S., Baik K.Y., Park J., Hong S. (2011). Controlling the growth and differentiation of human mesenchymal stem cells by the arrangement of individual carbon nanotubes. ACS Nano.

[26] Nayak T.R., Jian L., Phua L.C., Ho H.K., Ren Y., Pastorin G. (2010). Thin films of functionalized multiwalled carbon nanotubes as suitable scaffold materials for stem cells proliferation and bone formation. ACS Nano.

[27] Kitahara H., Kuboki Y., Takita H., Akasaka T., Watari F., Inoue N. (2010). Culture of ES cells and mesenchymal stem cells on carbon nanotube scaffolds. Nano. Biomed..

[28] Chao T.I., Xiang S., Chen C.S., Chin W.C., Nelson A.J., Wang C., Lu J. (2009). Carbon nanotubes promote neuron differentiation from human embryonic stem cells. Biochem. Biophys. Res. Commun..

[29] Yim E.K., Pang S.W., Leong K.W. (2007). Synthetic nanostructures inducing differentiation of human mesenchymal stem cells into neuronal lineage. Exp. Cell Res..

[30] McIlwain D.R., Berger T., Mak T.W. (2013). Caspase functions in cell death and disease. Cold Spring Harb. Perspect. Biol..

[31] Mooney E., Dockery P., Greiser U., Murphy M.M., Barron V. (2008). Carbon nanotubes and mesenchymal stem cells: biocompatibility, proliferation and differentiation. Nano Lett..

[32] Lacerda L., Bianco A., Prato M., Kostarelos K. (2006). Carbon nanotubes as nanomedicines: from toxicology to pharmacology. Adv. Drug Deliv. Rev..

[33] Chin S.F., Baughman R.H., Dalton A.B., Dieckmann G.R., Draper R.K., Mikoryak C., Musselman I.H., Poenitzsch V.Z., Pantano H.XieP. (2007). Amphiphilic helical peptide enhances the uptake of single-walled carbon nanotubes by living cells. Exp. Biol. Med..

[34] Nagai H., Okazaki Y., Chew S.H., Misawa N., Yamashita Y., Akatsuka S., Ishihara T., Yamashita K., Yoshikawa Y., Yasui H., Jiang L., Ohara H., Takahashi T., Ichihara G., Kostarelos K., Miyata Y., Shinohara H., Toyokuni S. (2011). Diameter and rigidity of multiwalled carbon nanotubes are critical factors in mesothelial injury and carcinogenesis. Proc. Natl. Acad. Sci. U. S. A..

[35] Giannona S., Firkowska I., Rojas-Chapana J., Giersig M. (2007). Vertically aligned carbon nanotubes as cytocompatible material for enhanced adhesion and proliferation of osteoblast-like cells. J. Nanosci. Nanotechnol..

[36] Lobo A.O., Antunes E.F., Machado A.H.A., Pacheco-Soares C., Trava-Airoldi V.J., Corat E.J. (2008). Cell viability and adhesion on as grown multi-wall carbon nanotube films. Mater. Sci. Eng. C.

[37] Lobo A.O., Corat M.A.F., Antunes E.F., Palma M.B.S., Pacheco-Soares C., Garcia E.E., Corat E.J. (2010). An evaluation of cell proliferation and adhesion on vertically-aligned multi-walled carbon nanotube films. Carbon.

[38] Lobo A.O., Marciano F.R., Ramos S.C., Machado M.M., Corat E.J., Corat M.A.F. (2011). Increasing mouse embryonic fibroblast cells adhesion on superhydrophilic vertically aligned carbon nanotube films. Mater. Sci. Eng. C.

[39] Huang H., Kamm R.D., Lee R.T. (2004). Cell mechanics and mechanotransduction: pathways, probes, and physiology. Am. J. Physiol., Cell Physiol..

[40] ZurNieden N.I., Kempka G., Ahr H.J. (2003). In vitro differentiation of embryonic stem cells into mineralized osteoblasts. Differentiation.

[41] Abdallah B.M., Jensen C.H., Gutierrez G., Leslie R.G.Q., Jensen T.G., Kassem M. (2004). Regulation of human skeletal stem cells differentiation by Dlk1/Pref-1. J. Bone Miner. Res..

[42] Sengupta P., Xu Y., Wang L., Widom R., Smith B.D. (2005). Collagen α1(I) gene (COL1A1) is represented by RFX family. J. Biol. Chem..

[43] McBeath R., Pirone D.M., Nelson C.M., Bhadriraju K., Chen C.S. (2004). Cell shape, cytoskeletal tension, and RhoA regulate stem cell lineage commitment. Dev. Cell.

[44] Settleman J. (2004). Tension precedes commitment–even for a stem cell. Mol. Cell.

[45] Kilian K.A., Bugarija B., Lahn B.T., Mrksich M. (2010). Geometric cues for directing the differentiation of mesenchymal stem cells. Proc. Natl. Acad. Sci. U. S. A..

[46] Li X., Liu H., Niu X., Yu B., Fan Y., Feng Q., Cui F.Z., Watari F. (2012). The use of carbon nanotubes to induce osteogenic differentiation of human adipose-derived MSCs in vitro and ectopic bone formation in vivo. Biometerials.

[47] Baik K.Y., Park S.Y., Heo K., Lee K.B., Hong S. (2011). Carbon nanotube monolayer cues for osteogenesis of mesenchymal stem cells. Small.

[48] Baktur R., Yoon S.H., Kwon S. (2013). Effects of multiwalled carbon nanotube reinforced collagen scaffolds on the osteogenic differentiation of mesenchymal stem cells. J. Nanomater..

[49] Chahine N.O., Collette N.M., Thomas C.B., Genetos D.C., Loots G.G. (2014). Nanocomposite scaffold for chondrocyte growth and cartilage tissue engineering: effects of carbon nanotube surface functionalization. Tissue Eng. Part A.

[50] Holmes B., Fang X., Zarate A., Keidar M., Zhang L.G. (2016). Enhanced human bone marrow mesenchymal stem cell chondrogenic differentiation in electrospun constructs with carbon nanomaterials. Carbon.

[51] Fabbro A., Prato M., Ballerini L. (2013). Carbon nanotubes in neuroregeneration and repair. Adv. Drug Deliv. Rev..

[52] Chen Y.S., Hsiue G.H. (2013). Directing neural differentiation of mesenchymal stem cells by carboxylatedmultiwalled carbon nanotubes. Biomaterials.

[53] Lee J.H., Lee J.Y., Yang S.H., Lee E.J., Kim H.W. (2014). Carbon nanotube-collagen three-dimensional culture of mesenchymal stem cells promotes expression of neural phenotypes and secretion of neurotrophicfactors. Acta Biomater..

